# Consumers’ coping strategies when they feel negative emotions in the face of forced deconsumption during the Covid-19 pandemic lockdowns

**DOI:** 10.3389/fpsyg.2022.1018290

**Published:** 2022-11-29

**Authors:** Myriam Ertz, Urvashi Tandon, Gautier G. Yao Quenum, Mohammed Salem, Shouheng Sun

**Affiliations:** ^1^LaboNFC, Department of Economics and Administrative Sciences, Université du Québec à Chicoutimi, Saguenay, QC, Canada; ^2^Chitkara Business School, Chitkara University, Patiala, Punjab, India; ^3^COlab, Cégep d’Alma, Saguenay, QC, Canada; ^4^Department of Administration and Finance Sciences, University College of Applied Sciences, Gaza, Palestine; ^5^School of Economics and Management, University of Science and Technology Beijing, Beijing, China

**Keywords:** deconsumption, COVID-19, coping, emotions, pandemic, lockdown, survey, questionnaire

## Abstract

**Introduction:**

This paper explores consumers’ coping strategies when they feel negative emotions due to forced deconsumption during the Covid-19 pandemic lockdowns.

**Methods:**

The tool used for data collection is the questionnaire. It was made using the LimeSurvey software. A total of 621 complete observations were analyzed.

**Results:**

The findings demonstrate that anger positively influences the activation of seeking social support, mental disengagement, and confrontive coping strategies. Besides, disappointment activates mental disengagement but only marginally confrontive coping and not behavioral disengagement. Furthermore, regret is positively related to confrontive coping, behavioral disengagement, acceptance, and positive reinterpretation. Finally, worry positively impacts behavioral disengagement, self-control, seeking social support, mental disengagement, and planful problem-solving.

**Discussion:**

The study’s originality lies in its investigation of consumers’ coping strategies when experiencing negative emotions due to forced deconsumption in the context of the Covid-19 pandemic.

## Introduction

At the end of 2019, the entire planet faced a crisis, first sanitary and then economic, called the Covid-19 pandemic. To curb the pandemic, government authorities worldwide have decreed health emergency measures such as lockdowns, widespread work stoppages, the closure of businesses selling non-essential goods and services, social distancing, or curfews (Mehrsafar et al., 2021; Warnock-Parkes et al., 2021).

These measures have led to a major upheaval in consumer consumption habits (Ivascu et al., 2022; Zhang and Li, 2022). Indeed, according to Colla (2020), except for fresh and natural products, the food sector has experienced considerable growth. In addition, the do-it-yourself (DIY), furniture and household appliances, electronics, video on demand (VOD), indoor games, home sports, and hygiene sectors experienced spectacular consumer success. On the other hand, sectors such as clothing and cosmetics have fallen sharply, leading to deconsumption (Colla, 2020). In addition, some well-established responsible consumption behaviors have also regressed, such as recycling, composting, sharing, or public transport (Trespeuch et al., 2020; Hansen et al., 2021).

Deconsumption may be defined from the consumer’s point of view as an individual’s behavior aimed at voluntarily reducing their consumption, at consuming less through the reduction of the sums spent, the reduction of the quantities consumed, or even the transfer of consumption. From certain products to others with better value for the consumer (de Lanauze and Siadou-Martin, 2013). But deconsumption, when forced, as in the context of the pandemic, can lead to negative consumer resentment.

In fact, this upheaval in consumption habits observed in individuals has generated various emotions, especially a “relative negative feeling of being less happy” (Martinelli et al., 2021, p. 18). In fact, “when lockdown measures were taken […] public response was marked with negative emotions” (Rodas et al., 2022, p. 323). In addition, to Garnefski and Kraaij (2006, 2018), these include, for example, anger, anxiety, depression, or stress. Therefore, how did consumers adapt to forced deconsumption during the Covid-19 crisis?

The rich theoretical framework on consumers’ adaptation to adverse events, also called “coping” (Lazarus and Folkman, 1984; Yi and Baumgartner, 2004; Duhachek, 2005; Gelbrich, 2009), can be advantageous in answering correctly to that critical question. Several studies, such as Yi and Baumgartner (2004) or Duhachek (2005), and Gelbrich (2009), studied the relationship between specific emotions and the activation of coping strategies. Given that Yi and Baumgartner’s (2004) research investigates how consumers manage stressful, emotional experiences in purchase-related situations, we shall retain this work as the theoretical framework of this study.

Consequently, this study answers the abovementioned question. It contributes fundamentally to the literature since it explores the coping strategies of consumers when they feel negative emotions related to forced deconsumption in a pandemic context and crisis context in general. The study also has practical, managerial, clinical, and applied importance, especially since the number of pandemics might potentially increase in the future, especially with climate change (Seo, 2021). These results enable managers and decision-makers to better anticipate consumer reactions and adjust their strategies and policies appropriately.

Our overall research objective is, therefore, to identify the different strategies that consumers have adopted in the context of the crisis to adapt to the upheaval in their consumption habits, and above all, to the forced deconsumption of specific goods and services.

To achieve this objective, we asked ourselves some specific research questions, namely:

1. How do consumers adapt to the anger felt following the forced deconsumption induced by the Covid-19 pandemic context?
2. How do consumers deal with disappointment after the forced deconsumption observed during the Covid-19 pandemic?
3. Faced with the regret felt following the involuntary consumption caused by the pandemic context of Covid-19, how do consumers adapt?
4. How do consumers adapt to the worry felt following the involuntary consumption caused by the context of the Covid-19 crisis?

The paper starts with a literature review on coping (Section “Literature review”) before presenting the theoretical and conceptual framework of the research (Section “Theoretical background and conceptual framework”). Then follow the research methodology (Section “Methodology”), the data analysis and results (Section “Analysis and results”), and the discussion of the results (Section “Discussion of the results”). Sections “Theoretical implications” and “Managerial implications” outline the implications for theory and practice, respectively. Section “Limitations and future research avenues” underscores the limitations of the research and their corresponding avenues for future research, while section 10 wraps up the paper with a short conclusion.

## Literature review

Yi and Baumgartner (2004) studied the adaptation of consumers to four negative emotions felt in a problematic purchasing situation: anger, regret, disappointment, and fear. Eight adaptation strategies emerge. Indeed, the consumer can get angry in front of a rude service provider. And to deal with anger, he can resort to confrontation (the consumer openly displays his dissatisfaction, defends his point of view, and tries to change the mind of the other party, the service provider, for example) or mental disengagement (the consumer moves on and avoids thinking about the situation). The consumer who feels disappointed because the products purchased do not live up to his expectations resorts to confrontation, mental disengagement, or behavioral disengagement (the consumer refuses any additional effort in the direction of the stressful situation). The consumer who feels he has made the wrong product choice and feels regret resorts to acceptance (the consumer accepts the unfavorable situation) or positive reinterpretation (the consumer finds a valid reason for the unfavorable situation and draws positive lessons). And finally, the consumer who is worried about the undesirable consequences linked to the purchase and consumption of a product resorts to the planned resolution of the problem (the consumer thinks about what can be done to manage the stressful situation, develops a plan of action, then takes the necessary steps to resolve the problem); seeking social support (the consumer seeks to discuss his feelings with a loved one in order to obtain comfort); self-control (consumers control and master their negative emotions) or even mental disengagement.

The interaction of negative emotions (fear and anger), coping strategies (acting out anger and psychological distance), and perceptions of information technology are examined by Zheng and Montargot (2022). The findings show that employees’ negative emotions (anger and fear) significantly and negatively affect how they perceive implementing a new reservation system by using coping strategies (i.e., venting anger and psychological distancing). Additionally, employees’ attitudes about using a cutting-edge reservation system positively impact their intention to do so.

Kwon and Kwak (2022) claim that the global COVID-19 pandemic drove the majority of sports leagues to postpone games in March and April 2020, leaving sports enthusiasts without any matches to watch. Their research investigated how sports fans assess stress and participate in coping strategies due to the global pandemic-related sports lockout. The findings demonstrated that anger, aggressiveness, and the desire for affiliation raised threat perceptions toward the COVID-19 lockout, which in turn had a substantial impact on coping strategies that were emotion-focused and disengaging.

The research by Bae (2022) investigated whether people’s coping strategies and the reasons they utilize social media serve as mediators between real COVID-19-related stress and the belief that doing so can be alleviated. The results revealed that the active coping strategies used by those experiencing COVID-19-related stress were more likely to be linked to informational and social interaction demands, leading people to attribute stress relief to social media use. Those under pressure were inspired to seek social engagement through the expressive support coping technique, which led people to believe that using social media to relax during the pandemic. By enabling people to lose themselves in social media activities and ignore negative thoughts related to the pandemic, emotional venting and avoidance coping strategies substantially influenced escape, social contact, and amusement seeking.

The paper of Kemp et al. (2021) attempts to investigate the distinctive emotional distress felt during the COVID-19 pandemic. It examines the function of fear and anxiety, what caused it, and how those emotions affected consumption as well as compliant and conformist behaviors. According to both exploratory and empirical studies, ruminative thoughts are positively correlated with fears and anxieties, but trust in leadership is inversely correlated with these emotions. Furthermore, large-scale purchases made following recommendations to stop the virus from spreading and regulate negative emotions *via* consumption were similarly linked to sentiments of fear and anxiety.

Satish et al. (2021) examined the Covid-19 pandemic-related changes in consumer behavior and purchasing patterns. Consumer stockpiling as a result of the COVID-19 pandemic has its own repercussions. The paper suggests that “minimalism in consumption” is crucial to preventing consumer greed. According to the study, customers’ buying habits will change if lockdowns are used in the future or during any other crisis. However, because they worry about the scarcity of necessities, consumers now have a hoarding mindset.

Nath et al. (2022) compared the consumers of India and Bangladesh and identified the existence of two emotion-based coping strategies, namely religiosity and social support. The authors further claimed that the COVID-19 pandemic strongly affected consumers’ general well-being. However, little is known about how the COVID-19 condition impacts consumer well-being and how subsistence consumers manage special tensions and well-being-related worries. The results show that subsistence consumers faced particular stressors and hardships during COVID-19, including unanticipated temporary financial difficulty, psychosocial stress, and stress connected to the market and consumption.

The study by Park et al. (2022) classified consumer groups based on their perceived negative emotions (i.e., anxiety, fear, depression, anger, and boredom). Four groups—anxiety, depression, anger, and indifference—were developed by clustering analysis. The study next looked at how each emotional group differs in its impact on the shopping-related motives (such as mood improvement, enjoyment of the shopping experience, socializing seeking, and self-control wanting) and actions (i.e., shopping for high-priced goods and buying bulk goods). The findings showed that all emotional groups had an impact on intentions for expensive buying as well as socializing seeking. However, depression and indifference are linked favorably to the need for social interaction and affect plans to buy in bulk. In addition, emotions other than anxiety impact mood enhancement and high-priced purchase intentions. Finally, anger influences intentions for bulk purchases and is linked to self-control striving.

Further, a study by Wang et al. (2017) highlighted that a negative encounter with a product or service disengages consumers, leaving the situation as an avoidance-focused coping strategy. Incongruity in emotions due to purchasing some faulty product leads to conflict in the minds of consumers, where they cogitate about whether they need the product, which in turn leads to negative behavior (Powers et al., 2019). In these situations, consumers regret and feel their responsibility toward purchase without careful consideration (Chan et al., 2017).

Moreover, in his study to better the theorization of coping strategies, Duhachek (2005) establishes links between eight coping strategies (active coping, rational thinking, positive thinking, emotional discharge, instrumental support, social support, avoidance, and denial) and some emotions related to the feeling of threat (fear, worry, threat, anxiety) and anger (anger and frustration). These include the link between negative emotions of threat and avoidance strategies, the link between threat and social support; but also, and the link between the threat and the active strategies (active adaptation, positive thinking, rational thinking).

Also, Gelbrich (2009) establishes a link between anger and the search for social support on the one hand and between anger and confrontation on the other hand. However, beyond this non-exhaustive list of studies on coping strategies and negative emotions, no previous (*a priori*) study has looked at consumers’ coping strategies when they experience negative emotions due to forced consumption in the context of a pandemic. The study by Cole et al. (2017) also insisted that a consumer may try to find some social support from his peers to arrive at emotional well-being if he receives a faulty product. But on the other hand, a study by Kim and Florack (2020) indicated that frequent social interactions could not provide stress relief during COVID-19, increasing emotional instability and triggering impulse buying. Authors further suggested that frequent interactions increased psychological emotions like fear and worry, affecting consumer behavior. Yuen et al. (2020) found an absence of knowledge during the pandemic as one of the factors that motivated them to shop more, feel secure, and relieve stress.

From the previous studies, it is evident that there is a semi-consensus on the importance of consumers’ coping strategies when they feel negative emotions in the face of forced deconsumption during the Covid-19 pandemic lockdowns.

## Theoretical background and conceptual framework

### Coping theory

According to Delelis et al. (2011), coping is part of a set of regulatory processes called affect regulation. Different approaches were proposed to explain that process, and among these different affect regulation approaches, the most popular and the subject of the most attention is Lazarus and Folkman’s (1984) regulation of stress or coping (Nicchi and Le Scanff, 2005). To them, the concept of stress regulation equates coping and refers to the process of managing negative emotions. More specifically, it uses various strategies to control or dissipate the stress caused by an unwelcome event. Consequently, Lazarus and Folkman’s (1984) conception will be retained for the remainder of this study. Besides, this study relates coping strategies to specific negative emotions, which corresponds to the research problem of this study: to explore the management of negative emotions under forced deconsumption.

Following Lazarus and Folkman (1984), coping is defined as “the dynamic use of cognitive and behavioral efforts to respond to external and internal demands assessed as exhausting or exceeding personal resources” (Nicchi and Le Scanff, 2005, p. 97). In other words, coping is an organized set of cognitive and behavioral efforts (strategies) that people make to anticipate and detect potential stressors or to manage (for example, prevent, minimize or control) the demand arising from transactions between themselves and their environment. The following sub-section delves deeper into those strategies.

### Classification of coping strategies

Coping strategies vary by author, but the most influential typology is the one developed by Lazarus and Folkman (1984) and Nicchi and Le Scanff (2005), which can either be problem-centered strategies or emotion-centered ones.

#### Problem-centered strategies

Problem-focused strategies involve efforts to manage or lessen the difficulty at the source of the stress. We distinguish, on the one hand, the preventive actions, which relate to the anticipation of the action and, therefore, to the reduction of the threat (gathering information, managing objectives, managing time, looking for solutions), and on the other, aggressive actions (confrontation; vindictive acts; vindictive complaints; expression of negative feelings; complaining), which eliminate or reduce the source of an existing difficulty.

Problem-focused strategies reduce the gap between the state of person-environment transactions and the desired (or hoped-for) state of these transactions. Furthermore, reducing this gap will curb the stress caused (Lazarus and Folkman, 1984; Nicchi and Le Scanff, 2005).

#### Emotion-centered strategies

Emotion-focused strategies (emotional management) are used when it is impossible to eliminate stress and involve regulating negative emotions resulting from the stressor. They do not impact the person-environment relationship but contribute to the individual’s well-being. These strategies involve predominantly physiological techniques, such as relaxation or cognitive efforts to change the meaning of the problem and reduce the threat (Lazarus and Folkman, 1984; Nicchi and Le Scanff, 2005). These five strategies centered on emotions, namely:

- Threat minimization: this technique gives little or no importance to the danger reflected in the stressful situation. For example, those who were called “conspiracy theorists” during the pandemic supported the position that the Covid-19 pandemic did not exist or was not as bad as announced in the media: the pandemic was more of a “plandemic” (Eberl et al., 2021). These individuals were reluctant to take barrier gestures or wear masks and, therefore, certainly experienced less stress.
- The positive reassessment of the situation consists of positively reinterpreting the situation with which one is confronted to dissipate the negative emotion one feels. For example, some observers (researchers, decision-makers, journalists, etc.) sought to positively reinterpret the pandemic as an opportunity to shift towards more sustainability and more responsible consumption (Trespeuch et al., 2021).
- Self-blame: recognizing one’s share of responsibility in a situation to forgive oneself and forget the situation and the stress that goes with it. Example: Gaétan buys a damaged product. He resolves not to go back to change it because he considers it his fault that he was not vigilant.
- Avoidance-flight from the stressor: fleeing or avoiding the stressor. For example, in front of impolite and aggressive agents enforcing mask-wearing and social distancing measures, a person is deterred from going to public places and prefers to stay home.
- Seeking social support: complaining about the situation to others in order to get their support. For example, a person who struggles with social distancing and lockdowns might find comfort and support in verbalizing his/her feelings to understand others.

**Figure 1:**
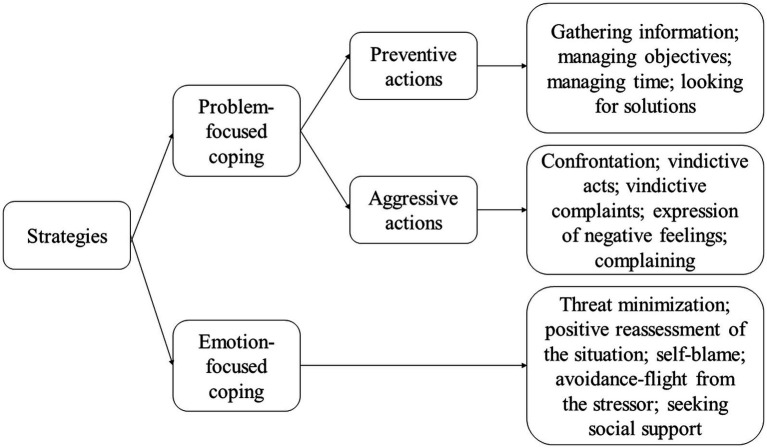
Coping strategies. Drawn using source data from Lazarus and Folkman (1984).

Figure 1 summarizes the types of coping strategies according to Lazarus and Folkman (1984).

**Figure 2:**
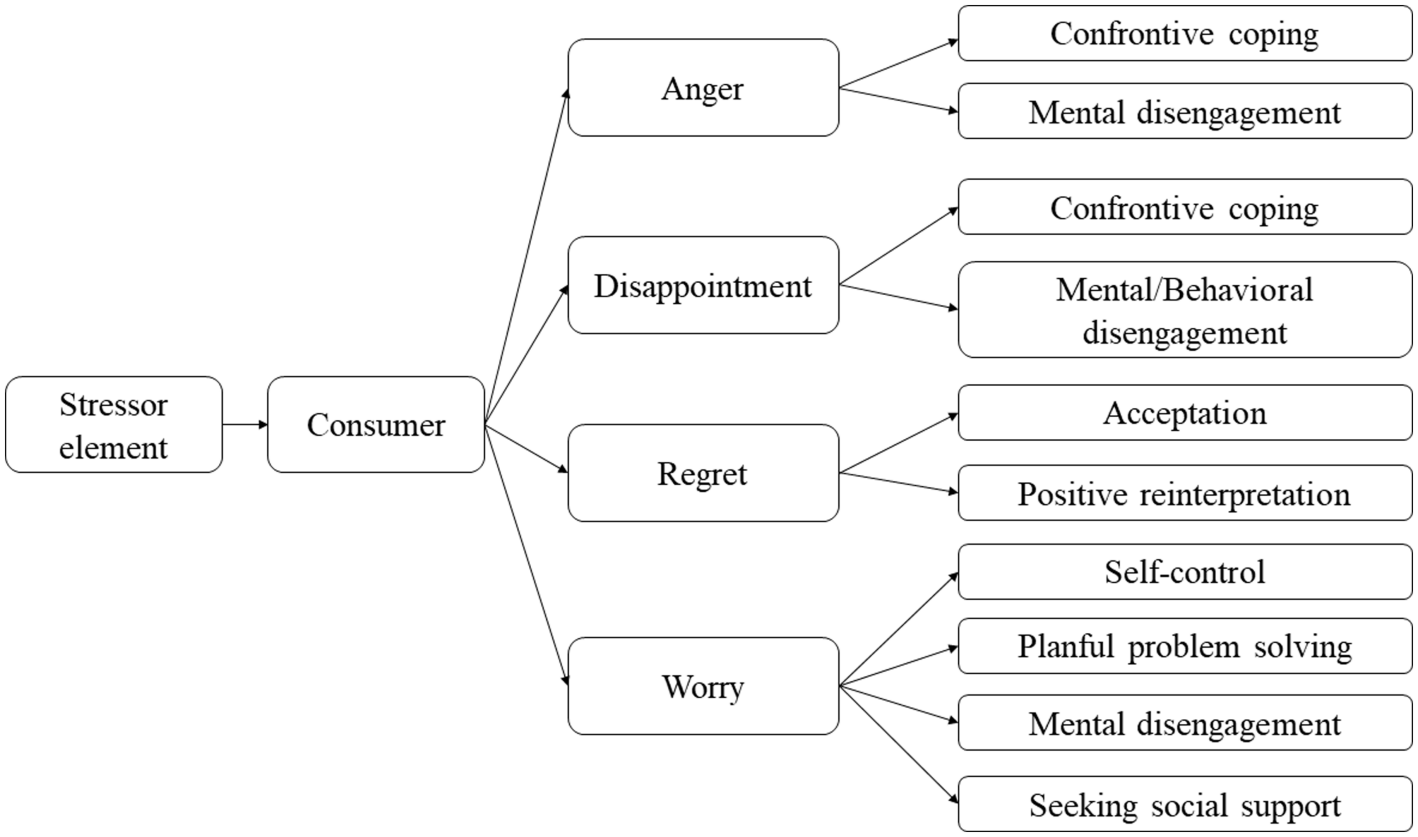
Consumers’ coping strategies. Drawn using source data from Yi and Baumgartner (2004).

### Consumers’ coping strategies

Several authors have studied coping strategies in consumption settings. Notably, Yi and Baumgartner (2004) identified eight strategies for managing negative emotions (i.e., anger, regret, worry, and disappointment) caused by destabilizing circumstances or events for the consumer. These are: “planned problem solving,”; “confrontation, “; “seeking of social support,”; “mental disengagement,”; “behavioral disengagement,”; “positive reinterpretation,”; “self-control,” and “acceptance of the problem.”

Indeed, when blame is assigned to another party and the situation is seen as changeable, as in the case of anger and disappointment, confrontation (the most important strategy in problem-based management and the least important in emotion-based management) is the most used. If the confrontation fails, the consumer uses mental disengagement.

When expectations are not met due to some circumstance (i.e., results-related disappointment), *mental* or *behavioral disengagement*, which is relatively unfocused on issues and emotions, is the most common coping strategy.

When consumers blame themselves for making the wrong choice and, therefore, experience regret, they tend to cope using *acceptance* and *positive reinterpretation*, which are less problem-focused and, therefore, more emotion-focused.

Finally, in cases of worry due to the prospect of future undesirable consequences, consumers refer to *planned problem-solving*, *seeking social support*, *self-control*, and *mental disengagement*.

The following diagram by Figure 2 summarizes the consumer’s strategies in a difficult situation and with negative emotions.

### Hypotheses development and conceptual framework

To Yi and Baumgartner (2004), consumers activate confrontation and mental disengagement strategies when they experience the emotion of anger. Duhachek (2005) abounds in the same direction and affirms that the emotion of anger, accompanied by a strong impression of effectiveness, leads to the adoption of active coping strategies (coping through action, rational thinking, and positive thinking) or expressive support (emotional relief, instrumental support, and emotional support). However, the emotion of anger associated with the impression of a low-efficiency level can lead to using these same strategies if the emotion of anger is very strong. Also, according to Duhachek (2005), the impression of a very low level of efficacy can lead angry consumers to adopt avoidance strategies (denial, avoidance). Gelbrich (2009) adds that anger associated with a low level of helplessness reinforces the activation of the strategy of vindictive complaint (act of confrontation) or seeking support (vindictive word of mouth) if the level of helplessness is high. Seeking social support could be an additional strategy for mental and behavioral disengagement. Therefore:

*H1a*: The emotion of anger has a positive influence on the activation of confrontive coping.

*H1b*: The emotion of anger has a positive influence on the activation of the mental disengagement strategy.

*H1c*: The emotion of anger has a positive influence on the activation of seeking social support.

Furthermore, according to Yi and Baumgartner (2004), disappointed consumers resort to confrontation, which is somewhat similar to the case of anger. And when the attempted confrontation strategies fail, consumers may resort to mental and behavioral disengagement. This leads us to postulate the following set of hypotheses:

*H2a*: The emotion of disappointment positively influences the activation of confrontive coping.

*H2b*: The emotion of disappointment positively influences the activation of the mental disengagement strategy.

*H2c*: The emotion of disappointment positively influences the activation of the behavioral disengagement strategy.

Moreover, the consumer who feels regret feels guilty for having transgressed his principles, standards, or values (Izard, 1977). This shows a complementarity between the emotion of regret and the emotion of guilt. Kubany and Watson (2003) demonstrate that the consumer who feels guilt engages in a positive reinterpretation of the events that led him to this feeling. Yi and Baumgartner (2004) validate the hypothesis that the consumer who feels regret tends to get over it by using acceptance and positive reinterpretation. Mattila and Ro (2008) claim that consumers who experience regret also employ confrontational strategies (direct complaint). However, for Le and Ho (2020), consumers who experience regret do not indulge in an immediate complaint; instead, they choose between either negative word-of-mouth or behavioral disengagement (they prefer to ignore the incident). Based on these developments, we build this other set of four hypotheses, namely:

*H3a*: The emotion of regret has a positive influence on the adoption of the confrontation strategy.

*H3b*: The emotion of regret has a positive influence on the adoption of the acceptance strategy.

*H3c*: The emotion of regret positively influences the adoption of the positive reinterpretation strategy.

*H3d*: The emotion of regret positively influences the adoption of the behavioral disengagement strategy.

In addition, for Yi and Baumgartner (2004), consumer refers to planned problem solving, seeking social support, self-control, and mental disengagement when experiencing worry. Moreover, Mercanti-Guérin (2008) thinks that worry can push the consumer to adopt a posture of avoidance and withdrawal with regard to the frightening situation, which relates to behavioral disengagement. Consequently, we decide to test the following hypotheses:

*H4a*: The emotion of worry positively influences self-control.

*H4b*: The emotion of worry positively influences mental disengagement.

*H4c*: The emotion of worry positively influences the activation of a planful problem-solving strategy.

*H4d*: The emotion of worry positively influences the activation of the social support seeking strategy.

*H4e*: The emotion of worry positively influences the activation of the search strategy of behavioral Disengagement.

**Figure 3:**
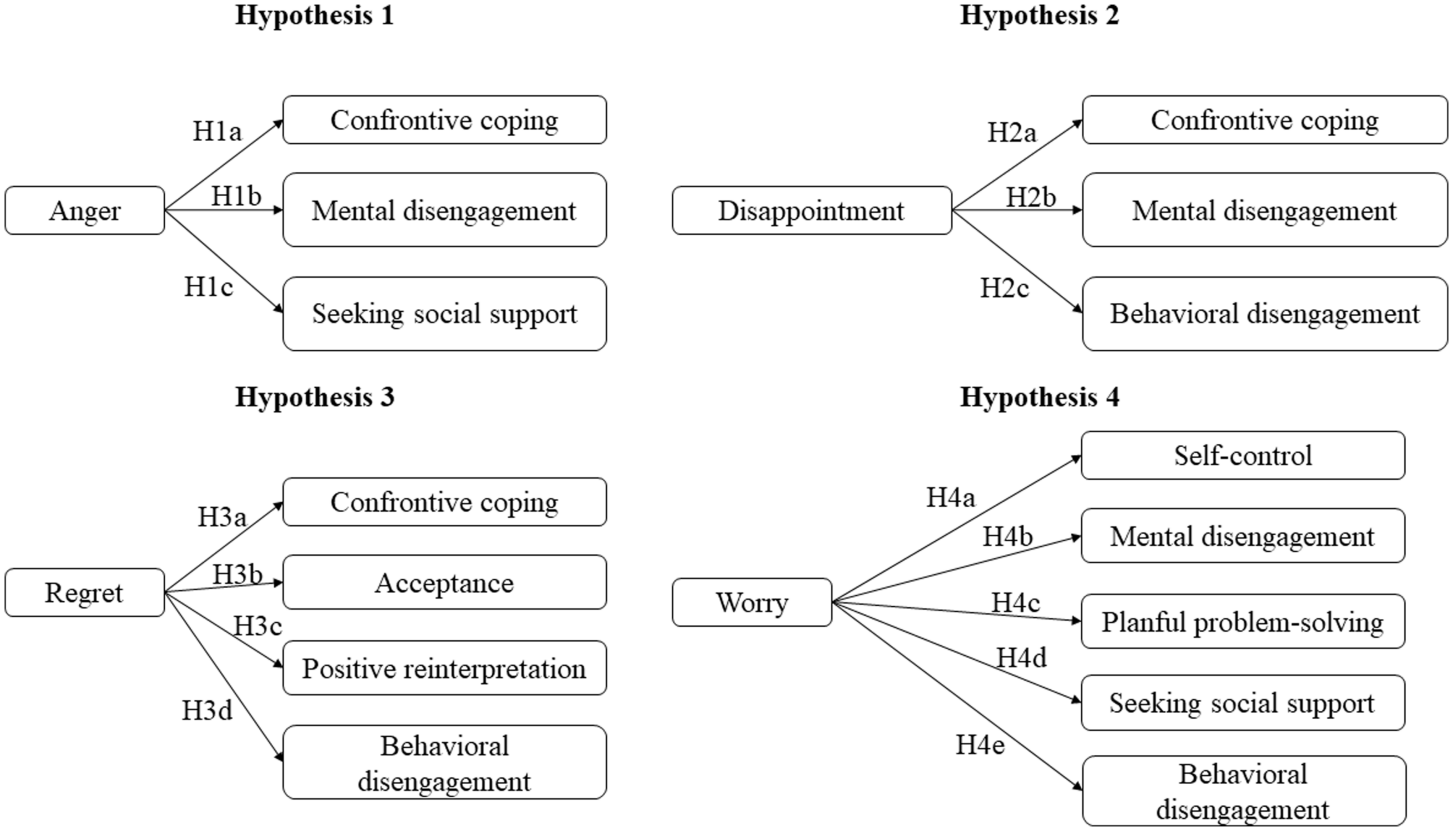
Conceptual framework.

The diagram in Figure 3 represents the conceptual model under study.

## Methodology

### Certificate of ethics

To ensure that our study meets all the ethical standards for research involving humans, we submitted our research project for approval to a university ethics certification committee. Our project has therefore been certified as compliant with ethical standards for research with human beings, and a certificate [no. 2021-554] has been awarded to us for this matter.

### Data collection tools

The data was collected by a survey questionnaire programmed with the LimeSurvey software. We used 5-point Likert scales (“1 = totally disagree,” “2 = Rather disagree,” “3 = Indifferent,” “4 = Rather agree,” and “5 = totally agree”) as instruments for gauging participants’ responses to each survey item.

### Questionnaire items

The questionnaire items are adapted from the measurement scale of eight coping strategies used in unpleasant buying situations by Yi and Baumgartner (2004). Table 1 shows the original items by Yi and Baumgartner (2004) and their adaptation to the current study.

**Table 1:** Items adapted to the study.

Items of Yi and Baumgartner (2004)	Adaptation to the current study
*Planful problem-solving*	
1. I thought about how I might best handle the problem	1. I often think about the best way to solve the problem (RP1)
2. I tried to come up with a strategy about what to do.	2. I try to find a strategy to solve the problem (RP2)
3. I thought about what steps to take to resolve the problem.	3. I think of steps to take to solve the problem (RP3)
4. I planned of action and followed it.	4. I have established a consumer action plan that I follow (RP4)
5. I knew what had to be done and I did it.	5. I know what I need to do to solve the problem (RP5)
*Confrontive coping*	
6. I tried to get the person responsible to change his or her mind.	6. I am trying to convince the government authorities to reopen the so-called non-essential businesses (CONF1)
7. I let the other person know how upset I was.	7. I often express my dissatisfaction to someone close to me (CONF2)
8. I presented my point of view and argued my case.	8. I often present my views on social media and advocate for my cause (CONF3)
9. I talked to someone to complain about the situation	9. I often complain to someone (CONF4)
10. I told someone about the problem and asked him or her to correct it.	10. I often entrust my case to someone to solve the problem (CONF5)
11. I expressed my feelings of displeasure without reservation.	11. I unreservedly express my dissatisfaction (CONF6)
*Seeking social support*	
12. I talked to someone about how I was feeling.	12. I talked to someone about how I was feeling (SS1)
13. I tried to get advice from someone about what to do.	13. I tried to get advice from someone about what to do (SS2)
14. I tried to get emotional support from family or friends.	14. I tried to get emotional support from family or Friends (SS3)
15. I asked someone I trust for help.	15. I asked someone I trust for help (SS4)
16. I discussed my feelings with someone.	16. I discussed my feelings with someone (SS5)
17. I talked to friends or relatives who know more about this than I do.	17. I talked to friends or relatives who know more about this than I do (SS6)
*Mental disengagement*	
18. I tried not to think about the situation.	18. I try not to think about the situation (DM1)
19. I wished that the situation would go away or somehow be over with.	19. I want to get rid of the situation so that I can consume again as before (DM2)
20. I tried to forget the whole thing.	20. I try to forget about everything related to the situation (DM3)
21. I wanted to turn the clock back.	21. I want to go back in time to before the pandemic (DM4)
22. I wished that I could escape from the situation.	22. I wish I could escape from the situation (DM5)
23. I wished I would not have to go through the situation.	23. I wish I did not have to go through this situation (DM6)
*Behavioral disengagement*	
24. I gave up the attempt to get what I wanted.	24. I abandon any desire to persevere in the exclusive purchase of so-called essential goods and services (DC1)
25. I acknowledged that the goal was beyond my reach.	25. It is out of my reach to buy exclusively so-called essential goods and services (DC2)
26. I could not go on, so I just quit trying.	26. I can no longer continue to consume exclusively so-called essential goods and services, so I have dropped everything (DC3)
27. I resigned myself to the fact that further efforts were futile.	21. For me, any additional effort is useless (DC4)
*Positive reinterpretation*	
28. I decided I had learned something from the experience.	28. Despite everything, I learn a lot from this experience (RPOS1)
29. I told myself the experience had taught me a valuable lesson.	29. I still draw valuable lessons from this experience (RPOS2)
30. I told myself this was a small price to pay for a lesson in life.	30. For me, this experience is a small price to pay for a life lesson (RPOS3)
31. I tried to look at the situation as an opportunity to learn something worthwhile.	31. This experience is an opportunity for me to learn something worthwhile (RPOS4)
*Self-control*	
32. I tried to keep my feelings to myself.	32. I camouflage my feelings with other feelings about the situation (MDS1)
33. I tried not to show other people how I really felt.	33. I try to contain my feelings about the situation (MDS2)
34. I tried to hold back my feelings.	34. I try to contain my feelings about the situation (MDS3)
*Acceptation*	
35. I realized I brought the problem on myself.	18. I tell myself that it is myself who created the situation (ACC1)
36. I accepted that it had happened and that it could not be changed.	36. I tell myself that this situation is inevitable (ACC2)
37. I learned to live with it.	37. I still learn to live with this new mode of consumption (ACC3)
38. I decided there was nobody to blame but me.	38. I only blame myself for this situation (ACC4)
39. I realized I had to accept the situation.	39. I tell myself that I have no choice but to accept the situation (ACC5)

Additional questions measure the level of emotions felt by the respondents. Negative emotions were not the subject of an experimental protocol in our study but were exclusively inspired by Yi and Baumgartner (2004), who had already done preliminary work on the four emotions (anger, disappointment, regret, worry) felt by consumers in difficult buying situations. Therefore:

•I feel angry that I have to buy less stuff.
•I feel disappointed that I have to buy less stuff.
•I feel regret because I have to buy less stuff.
•I feel worried because I have to buy less stuff.
- Finally, we added sociodemographic gender questions, including sex, age, gender, occupational status, marital status, and annual income.

### Sample size determination

When establishing the appropriate sample size, several factors must be considered, including the anticipated analytics. In our case, we plan to use factor analysis and structural equation modeling. We thus used a combination of approaches and techniques in order to triangulate for optimal sample size. First, a rule of thumb suggests at least ten respondents for questionnaire item, that is, a 10:1 ratio of respondents to item (Nunnally, 1978). Since we had 43 items except for five sociodemographic questions, this would have meant at least 430 respondents (or 480 with all survey questions included). Second, we turn to the literature that suggests a sample size independent of the number of measurement items. Usually, for factor analysis, a range of 200 to 300 observations is appropriate (Comrey, 1988; Guadagnoli and Velicer, 1988), but at least 300 to 450 is necessary to identify acceptable levels of comparability of patterns, while replication is necessary for sample sizes that are below 300 (Guadagnoli and Velicer, 1988). This is also in line with Clark and Watson’s (2016) suggestion of at least 300 observations after pre-test. Since a larger sample size is always better as it ensure more stable factor loadings, generalizable results, replicable factors and lower measurement errors (Comrey and Lee, 2013), we use Comrey and Lee’s (1992) graded scale (100 = poor; 200 = fair; 300 = good; 500 = very good; ≥ 1,000 = excellent). Although specific to scale measurement purposes, this scale provides numerical reference points to ensure a proper sample size. Since a sample above 500 respondents is deemed “very good,” and larger is always better for multivariate analysis (Osborne and Costello, 2004), we set the appropriate sample size at around 600 respondents.

### Recruitment of respondents

North American consumers aged 18 and over were recruited online on the Mturk platform. Despite its non-random sampling frame, MTurk has several desirable features: an integrated system of remuneration for participants, a large pool of participants, a simplified study design process, recruitment of participants, and data collection (Buhrmester et al., 2016). Besides, according to Buhrmester et al. (2016), compared to standard Internet samples, there is a slightly better demographic diversification of the MTurk respondents, speed in the recruitment process, lower cost of recruitment, and a collection of quality data. Within 1 month, we recruited 632 respondents, with 621 complete and 11 partial responses.

### Research procedures

The project has obtained a certificate of ethics [CER-2021-554] issued by the ethics committee from the university with which the authors are affiliated. Once recruited, the participants read an introduction to the study, which states the certificate of ethics, and then presents the research team and the study background and objectives. This section explains in detail that the Government has implemented several measures to curb the spread of the coronavirus. Some economic sectors (e.g., catering, events, tourism, sport/recreation) have been declared “non-essential” and have had to close their doors since December 25, 2020, or as early as September 28, 2020. As a consumer, this forces them to buy less than before by limiting themselves to “essential” goods and services (as defined by the Government’s “priority shopping list”). The text further states that the purpose of the study is to analyze how participants have dealt with these changes in their consumption patterns. Additional information was then provided to the respondents regarding the procedure (including the estimated completion time of 10 min), the risks and benefits of participating in the study, and different matters pertaining to confidentiality, retention of data, compensation, voluntary participation, and right of withdrawal of the study, and the responsibility of the principal investigator. The participants then provided their informed consent to participate in the study by ticking “yes” or “no.” Participants are then redirected to the questionnaire with mandatory fill-ins for all 48 questions. Since the survey was only for participants aged 18 and over, a screener question ensured that the participant was at least 18 years old. The questions relating to the emotion came first and were then followed by those on the coping strategies employed. After responding to the questions, a thank you page appeared on the screen and informed the participants that the survey was now over.

## Analysis and results

### Preliminary checks for data

The data was checked for quality and adequacy before applying statistical tools. Then, various preliminary statistical tests were applied to derive the results. First, the missing data were substituted with arithmetic mean, as suggested by Byrne (2013) and Shashi and Singh (2015). Further, the data were checked to detect the existence of common method bias (CMB). To this end, Harman’s single-factor test was performed (Harman, 1976). This procedure involves “constraining all the scale items into a single unrotated factor in exploratory factor analysis, with the assumption that the presence of CMB is indicated by the emergence of either a single factor or a general factor accounting for the majority of covariance among measures” (Podsakoff et al., 2003, p. 889). The recommended value is not more than 50% of the explained variance for the single-factor solution (Harman, 1976). The results indicated 32.25% for a single factor variance below the recommended value indicating that CMB is not present.

### Demographic profile

Table 2 presents the demographic profile of the sample. Of all the respondents, 64.73% were males, and 35.27% were females. The annual income of most of the respondents ranged between 50,000$–79,999$ (35.43%) and 20,000$–49,999$ (32.85). Undergraduates (45.57%) dominated the sample, followed by graduates holding a Master’s degree (21.90%) and a Professional degree (13.37%). The vast majority of respondents were full-time employees (79.87%) and aged 25 to 44 (72.8%).

**Table 2:** Demographic Profile.

Demographic characteristics, *N* = 621	Frequency	Percentage
*Gender*		
Male	402	64.73
Female	219	35.27
*Annual income*		
Less than 20,000$	80	12.88
20,000$-49,999$	204	32.85
50,000$-79,999$	220	35.43
80,000$-119,000$	92	14.81
1,200,004$ and more	25	4.03
*Education qualification*		
Primary	15	2.42
Secondary	31	4.99
College	55	8.86
Undergraduate, e.g., certificate, bachelor	283	45.57
University graduate, e.g., DESS, Master’s Degree	136	21.90
Professional degree, e.g., MD, DOS, DVM, LLB	83	13.37
University postgraduate, e.g., Doctorate, Ph.D	18	2.90
*Occupancy status*		
Student	7	1.13
Full-time employee	496	79.87
Part-time employee	43	6.92
Unemployed	10	1.61
Homemaker	21	3.38
Self-employed	38	6.12
Retired	6	0.97
*Age*		
18–24	60	9.7
25–44	452	72.8
More than 44	109	17.5

### Measurement model

Confirmatory Factor Analysis (CFA) was performed to assess the reliability and validity of the data and confirm the theoretically grounded model reflecting postulated relationships between exogenous and endogenous constructs, Confirmatory Factor Analysis (CFA) was performed (Mueller and Hancock, 2001, p. 5240). To estimate the convergent validity, the standardized loadings of the constructs and the average variances extracted (AVEs) were considered (Hair et al., 2011). Standardized loadings of 0.6 or higher suggest that items exhibit validity (Kline, 2005). AVE values above 0.5 indicate adequate convergent validity (Fornell and Larcker, 1981; Bagozzi et al., 1991). Internal consistency (i.e., reliability) was addressed by computing composite reliability (CR). 0.7 signals an acceptable internal consistency (Fornell and Larcker, 1981; Castagna et al., 2020).

Table 3 shows the measurement model results. A few scale items, such as RP4 for Planful problem solving, DM2 and DM4 for Mental Disengagement, RPOS3 for Positive Interpretation, MDS1 for Self-control, as well as ACC2 and ACC3 for Acceptance were removed due to low factor loadings. The standardized item loadings lay between 0.600 and 0.824, thus exceeding the recommended minimum value of 0.60 (Kline, 2005; Hair et al., 2011). The critical ratio values of all the scale items were above 1.96, suggesting a normal data distribution (Byrne, 2013). These results indicate the existence of convergent validity. Composite reliabilities (CRs) of the variables lay between 0.711–0.89 and are above the recommended value of 0.7, reflecting good internal consistency of the factors. The Average Variance Extracted (AVE) of each construct was above the recommended value of 0.5 and lay between 0.507–0.656, indicating that all constructs exhibit convergent validity (Fornell and Larcker, 1981).

**Table 3:** Measurement model.

		Std. Estimate	Std. Error	Critical ratio	Average variance extracted	Composite reliability	Cronbach’s alpha
Planful problem solving	RP1	0.773			0.524	0.814	0.813
	RP2	0.707	0.058	16.743			
	RP3	0.742	0.059	17.580			
	RP5	0.669	0.056	15.830			
Confrontive coping	CONF1	0.744			0.507	0.860	0.861
	CONF2	0.626	0.050	15.456			
	CONF3	0.762	0.052	19.094			
	CONF4	0.710	0.053	17.676			
	CONF5	0.757	0.051	18.974			
	CONF6	0.664	0.052	16.463			
Seeking social support	SS1	0.745			0.574	0.890	0.889
	SS2	0.774	0.060	19.326			
	SS3	0.707	0.059	17.526			
	SS4	0.786	0.062	19.658			
	SS5	0.741	0.06	18.439			
	SS6	0.789	0.059	19.742			
Mental disengagement	DM1	0.719			0.507	0.753	0.745
	DM3	0.791	0.059	17.550			
	DM5	0.614	0.055	13.942			
Behavioral disengagement	DC1	0.743			0.513	0.807	0.800
	DC2	0.736	0.056	18.602			
	DC3	0.774	0.059	19.670			
	DC4	0.600	0.065	14.925			
Positive reinterpretation	RPOS1	0.700			0.529	0.771	0.769
	RPOS2	0.704	0.073	14.721			
	RPOS4	0.776	0.078	15.660			
Self-control	MDS2	0.780			0.553	0.711	0.793
	MDS3	0.705	0.056	17.516			
Acceptance	ACC1	0.774			0.656	0.792	0.789
	ACC4	0.844	0.053	22.033			

Discriminant validity was measured by calculating the AVE’s square root, which ranges between 0.712 and 0.809 (see the diagonal values in Table 4). All these values were above the inter-item correlations (see the off-diagonal values in Table 4), meeting the discriminant validity criteria (Hair et al., 2011).

**Table 4:** Correlation matrix.

	RP	CONF	SS	DM	DC	RPOS	MDS	ACC
RP	**0.723**							
CONF	0.478^**^	**0.712**						
SS	0.550^**^	0.689^**^	**0.757**					
DM	0.382^**^	0.627^**^	0.553^**^	**0.712**				
DC	0.348^**^	0.618^**^	0.630^**^	0.624^**^	**0.716**			
RPOS	0.537^**^	0.348^**^	0.484^**^	0.342^**^	0.303^**^	**0.727**		
MDS	0.412^**^	0.561^**^	0.519^**^	0.541^**^	0.587^**^	0.476^**^	**0.730**	
ACC	0.365^**^	0.737^**^	0.647^**^	0.589^**^	0.753^**^	0.325^**^	0.607^**^	**0.809**

^**^Correlation is significant at the 0.01 level (2-tailed). RP, Planful problem solving; CONF, Confrontation coping; SS, Social Support; DM, Mental Disengagement; DC, Behavioural Disengagement; RPOS, Positive Reinterpretation; MDS, Self-Control; ACC, Acceptance. The values in the diagonal refer to the square roots of the average variance extracted pertaining to the corresponding variable.

### Structural model

After ascertaining the satisfactory factor structure, the proposed hypotheses positing relationships between dependent and independent variables were tested using structural equation modeling (SEM). Table 5 provides the results of the structural model. Model fit indices indicated a good model fit (CMIN/df = 4.22, GFI = 0.985, NFI = 0.989, CFI = 0.991, TLI = 0.960, IFI = 0.982, RMSEA = 0.071).

**Table 5:** Structural model.

			Std. Estimate	Std. Error	Critical ratio	*P*	Result
Anger	→	Confrontive coping	0.142	0.026	3.833	***	Supported
Anger	→	Mental disengagement	0.177	0.029	3.905	***	Supported
Anger	→	Seeking social support	0.184	0.024	4.833	***	Supported
Disappointment	→	Confrontive coping	0.060	0.025	1.713	0.087	Not-Supported
Disappointment	→	Mental disengagement	0.156	0.032	3.269	0.001	Supported
Disappointment	→	Behavioral disengagement	0.033	0.028	0.887	0.375	Not-Supported
Regret	→	Confrontive coping	0.199	0.026	5.497	***	Supported
Regret	→	Acceptance	0.14	0.034	3.874	***	Supported
Regret	→	Positive reinterpretation	0.101	0.02	3.865	0.004	Supported
Regret	→	Behavioral disengagement	0.177	0.026	4.912	***	Supported
Worry	→	Self-control	0.391	0.023	11.286	***	Supported
Worry	→	Mental disengagement	0.261	0.031	5.420	***	Supported
Worry	→	Planful problem solving	0.243	0.019	6.829	***	Supported
Worry	→	Seeking social support	0.315	0.026	7.557	***	Supported
Worry	→	Behavioral disengagement	0.400	0.029	9.720	***	Supported

*** *p* < 0.01.

The impact of anger was empirically validated on confrontive coping, mental disengagement, and seeking social support. Seeking social support was the most strongly impacted by anger (β = 0.184, *p* ≤ 0.001), followed by mental disengagement (*β* = 0.177, *p* ≤ 0.001). Confrontive coping (*β* = 0.142, *p* ≤ 0.001), though significant, was less influenced by anger than mental disengagement and seeking social support. Collectively, these results lend support to H1a–c.

Disappointment significantly influenced mental disengagement (*β* = 0.156, *p* = 0.001), supporting H2b. This indicates that disappointed consumers tend to escape from the situation and try to forget the situation of forced deconsumption. Surprisingly, the emotion of disappointment had only a marginal impact on confrontive coping (*β* = 0.060, *p* = 0.087) and did not significantly impact behavioral disengagement (*β* = 0.033, *p* = 0.375), so H2a and H2c are not supported.

The impact of regret was assessed on confrontative coping, acceptance, positive reinterpretation, and behavioral disengagement. Regret strongly influenced confrontive coping (*β* = 0.199, *p* ≤ 0.001) and behavioral disengagement (*β* = 0.177, *p* ≤ 0.001), thus supporting H3a and H3d. Regret also influenced acceptance (*β* = 0.140, *p* ≤ 0.001). Albeit significant, positive reinterpretation was the least impacted by regret (*β* = 0.101, *p* = 0.004) compared to other coping strategies. These results collectively support H3b and H3c.

The effect of worry was estimated on self-control, mental disengagement, planful problem-solving, seeking social support, and behavioral disengagement. Among all, behavioral disengagement (*β* = 0.400, *p* = 0.000) and self-control (*β* = 0.391, *p* ≤ 0.001) were the most impacted by worry. Furthermore, seeking social support (*β* = 0.315, *p* ≤ 0.001), mental disengagement (*β* = 0.261, *p* ≤ 0.001), and planful problem-solving (*β* = 0.243, *p* ≤ 0.001) were also significantly influenced by the emotion of worry. Therefore, H4a–e are supported.

## Discussion of the results

Coping strategies are derived from work on reaction to stress (Lazarus and Folkman, 1984) and, as such, are highly relevant to the study of the consumers’ responses to the key stressors of the Covid-19 pandemic and ensuing lockdowns. Using Yi and Baumgartner’s (2004) theoretical framework on coping strategies under difficult purchasing situations, this research investigates consumers’ coping strategies when they feel negative emotions in the face of forced deconsumption during the Covid-19 pandemic lockdowns. In fact, Yi and Baumgartner’s (2004) study findings are continually used as a framework of reference to draw connections between coping mechanisms and negative emotions in the circumstances involving purchases (Jun and Yeo, 2012; Zheng and Montargot, 2022).

The results of Zheng and Montargot (2022) show how critical it is to consider unfavorable feelings while adopting IT innovations. Additionally, the model created in this study supports that, compared to a valence-based approach, an appraisal tendency approach better defines the circumstances in which various emotions are activated to anticipate and explain how emotions connect to IT usage through adaption actions. Kwon and Kwak (2022) offer factual proof of how sports fans react to the pandemic-related sports lockdown and deal with the unusual circumstances. By classifying consumers according to their psychological tendencies, it may be possible to anticipate which sports fans would participate in coping strategies. According to Bae (2022), communicators can better understand how users can encourage people to cope with stress by providing people with more effective social media, which will lead to stress reduction and improved well-being by understanding how stress-induced coping strategies influence people’s specific motivations and reduce users’ stress levels. The study of Kemp et al. (2021) sheds fresh light on what causes fear and anxiety during pandemics and explores how these emotions affect consumption as well as conformity and compliance behaviors. According to Satish et al. (2021), a situational impact of the pandemic has been a sharp shift in consumer behavior. Each crisis has a unique impact on consumer behavior. In this study, Covid-19 was taken into account when analyzing fear, greed, and anxiety. On the other hand, the research aims to make reasonable inferences based on customers’ experiences during the lockdown. Based on the data of Nath et al. (2022) study, which is based on the appraisal theory of stress, reveals that religion and social support, two emotion-focused coping strategies, coexist in India and Bangladesh and work together to help people overcome their worries about their well-being. The severe effects of the COVID-19 pandemic on customers who are socioeconomically subsistence may thus be of special importance to managers and policymakers. In pandemic scenarios like the present COVID-19 issue, the study of Park et al. (2022) helps practitioners and academics better understand how individuals manage their negative emotions by engaging in retail therapy.

The results demonstrate that anger positively influences the activation of seeking social support, mental disengagement, and confrontive coping, and this finding is in sync with the previous study by Stone et al. (2003). That angry customers mainly seek social support in the face of anger underscores the generic importance of social ties and relationships in the wake of crises (Hobfoll et al., 1986; Stone et al., 2003). Mental disengagement’s secondary importance could be explained by the fact that this strategy may appear after consumers’ original outpouring of anger dissipates or after the situation becomes unchangeable. Interestingly and counter-intuitively, confrontive coping, which places a higher emphasis on (aggressive) problem-solving than on emotions (Lazarus and Folkman, 1984), was the last coping mechanism employed by customers to control their anger. This can be explicable by the specific nature of the Covid-19 crisis, during which consumers were confined at home and could not easily confront those they deemed responsible for the situation. On the other hand, they could communicate well with other people. In fact, Internet communications boomed during that period (Abir et al., 2021; Wong et al., 2021), hence the relative prevalence of seeking social support over confrontation.

Second, the results underline that the emotion of disappointment has a milder effect on coping strategies. These results contradict the previous studies by Duhachek (2005) and Gelbrich (2009), where disappointed consumers restrain themselves from any purchase. In contrast to angry consumers who resort to a broader range of coping strategies, disappointed ones tend to recourse exclusively to mental disengagement. Although the impact on confrontive coping was marginally significant, a parallel between mental disengagement and confrontive coping can be drawn. More specifically, the observation that disappointment might occasionally be person-related could trigger confrontational coping (see van Dijk et al., 2019). In fact, although the issue may be context-based (e.g., government decrees and stores adapting to new regulations), consumers who are disappointed have a strong tendency to hold another party (such as the marketer) accountable for the fact that their expectations were not met, even when the exchange partner was not directly at fault for the issue (Yi and Baumgartner, 2004). This can be because consumerism has pushed people to stand up for their rights when confronted with a poor product or service experience (Khalil et al., 2021).

Additionally, dissatisfaction and mental disengagement are linked. For example, when consumers blamed the disconfirmation on impersonal circumstances (i.e., when the disappointment was result-related) and believed that nothing could be done to remedy the situation, dissatisfied customers may use mental disengagement (Jung and Park, 2018). Future studies should make a clearer distinction between the two types of disappointment, especially in light of how differently they affect coping mechanisms.

Third, similarly to anger, the emotion of regret arising from forced deconsumption due to the Covid-19 pandemic activates a broad range of coping strategies. These include, respectively, confrontive coping, behavioral disengagement, acceptance, and positive reinterpretation. Acceptance and positive reinterpretation were utilized by customers who felt remorse in dealing with their emotional condition. Both coping mechanisms place a strong emphasis on emotions over problems. However, in contrast to Yi and Baumgartner’s (2004) findings, the lower impact of regret on both strategies indicates that regretful customers are slightly less likely to make an effort to regulate or alter their feelings as well as adjust to the circumstance in the case of forced deconsumption due to the Covid-19 pandemic. Instead, confrontive coping and behavioral disengagement were more prevalent, which could be explained by consumers’ longing for how things were before the pandemic outbreak, before the mandates, and before the lockdowns. Gittings et al. (2021) underscore this by emphasizing how consumers felt that their lives got “stuck” (p. 947) during the lockdowns. In fact, many found it preferable to “get back to normal” (NHS, 2021) instead of going further into the “new normal” (Emanuel et al., 2022, pp. 211–212).

Fourth, the findings show that worry positively influences, by order of importance, behavioral disengagement, self-control, seeking social support, mental disengagement, and planful problem-solving. As predicted by Yi and Baumgartner’s (2004) framework, worry produced the broadest range of coping mechanisms compared to the other emotions. Consumers who experienced anxiety disengaged behaviorally, exercised self-control, sought social support, disengaged mentally, and solved problems planfully. Worry is a response to the possibility of an unfavorable future with little control and predictability. Consequently, a rational, problem-focused approach seems inappropriate for dealing with such emotion. Instead, worried consumers seem to predominantly recourse to emotion-based coping approaches such as avoidance-flight from the stressor and self-mastery, according to Lazarus and Folkman’s (1984) typology. More specifically, they adjust to the circumstance and regulate the feeling *via* behavioral detachment – and, to a lesser extent, mental disengagement - and self-control. Yet, as underscored by Yi and Baumgartner (2004), despite unpredictability, the results further show that worry also entails problem-based strategies, especially seeking social support and planful problem-solving.

## Theoretical implications

This study contributes to the literature by exploring how consumers cope with four negative emotions from forced deconsumption amid the Covid-19 pandemic outbreak and ensuing lockdowns. As such, it contributes to advancing the literature on consumer adaptation and coping strategies.

It has been argued so far in the literature (e.g., Yi and Baumgartner, 2004; Duhachek, 2005) that in the event of confrontation and social support failure, angry consumers adopt disengagement (avoidance) strategies. We have demonstrated that in the specific context of lockdowns, angry consumers – due to their inability to access their leaders and go out of their homes - resort slightly more to social support seeking and avoidance through mental disengagement than to confrontational strategies.

Furthermore, past research (e.g., Yi and Baumgartner, 2004; Mattila and Ro, 2008) suggested that when feeling disappointed, consumers can adopt confrontative strategies, avoidance, and, albeit more marginally, seeking social support. This study indicates that consumers disappointed by forced deconsumption in a crisis context preferred avoidance strategies and mental disengagement. The lack of impact on either confrontation or behavioral disengagement can be related to the absence of access to authorities and, syllogistically, the incapacity to avoid them.

Kubany and Watson (2003) suggest that consumers who feel regret adopt positive reinterpretation strategies. Similarly, Yi and Baumgartner (2004) argue that in the event of regret, consumers use positive reinterpretation and acceptance strategies. As for Mattila and Ro (2008), in the event of regret, the consumer can use confrontation to search for social support. If those strategies all fail, the regretful consumer may decide to simply ignore the incident, so he behaviorally disengages from it (Le and Ho, 2020). Our study has shown that while disappointment does not produce confrontive coping, and anger activates that strategy but slightly less automatically than others, regret is the most conducive to confrontation (*cf.* Mattila and Ro, 2008) and behavioral disengagement. Nostalgic feelings which create retro perspectives (Hallegatte et al., 2018) by thinking about the days before the pandemic (and how better they were for some people [Gittings et al., 2021]) appear as a stronger drive for aggressive problem-based coping than disappointment or even anger. The study also confirms the emergence of acceptance and positive reinterpretation, in line with past research (e.g., Kubany and Watson, 2003; Yi and Baumgartner, 2004).

In the event of worry, consumers may activate several active strategies (planned resolution and social support) and emotion-focused strategies (mental disengagement and self-control; Yi and Baumgartner, 2004). To Mercanti-Guérin (2008), consumers tend to adopt a posture of avoidance and withdrawal regarding the frightening situation. Our study specifies and complements Mercanti-Guérin’s (2008) work in that, overall, avoidance is predominant in the form of behavioral disengagement. In line with Yi and Baumgartner (2004), this strategy is followed by self-control, and both are emotion-focused strategies, while other strategies are more problem-focused (i.e., seeking social support and planful problem solving).

This research draws on Yi and Baumgartner’s (2004) study as the theoretical framework in this paper. However, since their study investigates how consumers cope with stressful, emotional experiences in generic purchase-related situations, their results remain general and *a priori* inapplicable to extreme consumption events such as forced deconsumption induced by the Covid-19 pandemic. In fact, while confrontive coping appeared to be the most prominent when feeling anger and disappointment (Yi and Baumgartner, 2004), in our study, this strategy came only third after social support seeking and mental disengagement for anger and did not even constitute an outcome of disappointment. This absence of immediate confrontive coping in response to anger or disappointment may be due to the fact that in contrast to conventional purchase situations where consumers may attribute the issue related to the negative emotion to another person, the responsibility for Covid-19-related policies and measures (e.g., obligation to purchase essential products and services) did not only involve a single individual (e.g., employee, franchisee) or specific retails chains or brands, but rather numerous agents, including municipal authorities, provincial and federal/national governments, and even supranational entities (e.g., WHO). This is also manifest in mental disengagement - a strategy appearing second after an anger outburst [anger] or when the situation seems unchangeable [disappointment] (Yi and Baumgartner, 2004).

However, while regret generated two emotion-based strategies, such as acceptance and positive reinterpretation in Yi and Baumgartner (2004), our study showed that this emotion triggers aggressive problem-based coping through confrontive strategies and behavioral disengagement. In sum, nostalgic feelings of regret seem more conducive to aggressive problem-based coping and, to a lesser extent, acceptance and positive reinterpretation, possibly when the situation appears unchangeable.

Finally, we concur with Yi and Baumgartner (2004) that worry generates the most diverse assortment of coping strategies. Yet, those found by Yi and Baumgartner (2004) slightly differ from ours. Behavioral disengagement appeared first, although Yi and Baumgartner (2004) identified this as a non-viable strategy because worry concerns the prospect of undesirable future events. In our case, behavioral disengagement with a merchant might have consisted in using online commerce, which notably boomed during that period since access to stores for non-essential goods was forbidden. However, although not necessarily in the same order, our study matches Yi and Baumgartner’s (2004) in that the following strategies consisted mainly of self-control, seeking social support and planful problem-solving.

## Managerial implications

Under extreme situations such as forced deconsumption due to the Covid-19-related lockdowns and closure of non-essential businesses, consumers who experience anger and/or regret are the most likely to resort to direct confrontation with whomever they deem responsible for the situation, including retailers and business owners. They are more reluctant, less collaborative, and engage in attitudes of persuasion and retaliation. Hence, they require particular attention. The desire to deal with the restrictive situation means that this type of consumer would likely collaborate if they are made aware and supported. According to Bonifield and Cole (2007), recovery efforts that attenuate anger decrease consumer retaliatory attitudes. Therefore, we recommend that marketers, producers, and business leaders complement their conventional products and/or services with additional or complementary ones daringly. For example, two products for one, small gifts (e.g., pens) and notably products that specifically answer the consumer’s needs during the crisis), or even a thank-you note underscoring the retailer’s gratitude to the consumer for supporting local businesses. These may constitute forms of recovery to make up for the situation. Also, resistant products over time should be offered to facilitate long-term use and therefore reduce the purchase and excessive consumption of goods. Angry and worried consumers will seek social support, and hence human presence, be they clerks, store managers, and overall staff, will act as reassuring reference points for them. It will be necessary for employees to be good ambassadors of the brand in that process. Worried customers, in particular, will necessitate assistance in jugulating their emotions as they resort primarily to emotion-based strategies. Although disappointed consumers are least likely to activate a broad array of coping strategies, and if they do, they will seek to disengage from the situation mentally, managers may still be able to assist those consumers while also caring for angry and worried consumers as well. Conducting “business as usual” and displaying minimal references to the crisis is particularly suitable.

## Limitations and future research avenues

Although conducted to the best of our abilities, this study is not without limitations. It investigates four negative emotion variables without using control variables to check whether consumers felt other emotions. In addition, although the context of the pandemic had a strong effect, the emotions felt by consumers may be linked to other factors such as the virus, unemployment, bankruptcy, indebtment, lockdown, and so on, but this has not been controlled for in this study. Additional studies might therefore investigate the effect of such variables and of other unrelated variables by using them as control variables, for example. Besides, future research could explore additional emotions using Duhachek’s (2005) typology (e.g., fear, worry, threat, anxiety, anger, and frustration) and control for specific adverse outcomes of the lockdowns for respondents. Moreover, since the study relies on self-reported data, doubts about emotions felt during lockdowns and forced deconsumption could have caused bias in the data collected. However, we are confident that a traumatic situation such as a quasi-worldwide lockdown is so exceptional and unprecedented that it marks individuals and imprints their memory. In fact, several researchers diagnosed the Covid-19 pandemic as a “traumatic stressor” (Bridgland et al., 2021), which even caused post-traumatic stress disorders (PTSD) symptomatology and various other psychological problems worldwide due to the psychological distress caused by the Covid-19 emergency (Alshehri et al., 2020; Forte et al., 2020; Liang et al., 2020). Besides, the analytical design reinforces the robustness of the results by allowing the direct examination of negative emotion variables and their link with coping strategies. Another limitation of this study is that we did not use control for “internet purchase.” While it is clear that consumers have deconsumed by buying products in stores, they might have compensated for this forced deconsumption by shopping online. However, suppose we start from the premise that Internet shopping might dampen negative feelings. If the study design can still capture negative emotions and their significant effect on coping, then the design and related results are rather conservative and should increase trust in the findings. Additional studies using this variable as a control or as a group differentiator might nonetheless find possibly stronger effects among consumers who did not purchase online.

## Data availability statement

The raw data supporting the conclusions of this article will be made available by the authors, without undue reservation.

## Ethics statement

The studies involving human participants were reviewed and approved by Comité d’éthique de la recherche, Université du Québec à Chicoutimi. The patients/participants provided their written informed consent to participate in this study.

## Author contributions

ME and GQ devised the project, the main conceptual ideas, and the proof outline, collected the data, and conceived and planned the survey. GQ and SS performed the preliminary set of analyses. UT curated the data, performed the analysis, and reported the results. ME wrote the manuscript in consultation with UT, MS, and SS, and supervised and funded the project. MS reviewed the manuscript. SS formatted the manuscript with the help of ME. All authors contributed to the article and approved the submitted version.

## Funding

This research has benefitted from the Covid 2020–2021 supplement provided by the Social Sciences and Humanities Research Council (SSHRC) of Canada and is related to another grant in consumption studies received by the first author (grant number 430-2018-00415).

## Conflict of interest

The authors declare that the research was conducted in the absence of any commercial or financial relationships that could be construed as a potential conflict of interest.

## Publisher’s note

All claims expressed in this article are solely those of the authors and do not necessarily represent those of their affiliated organizations, or those of the publisher, the editors and the reviewers. Any product that may be evaluated in this article, or claim that may be made by its manufacturer, is not guaranteed or endorsed by the publisher.
